# Severe upper airway dysfunction in *GNAO1*-related disorders

**DOI:** 10.1186/s13052-025-02150-0

**Published:** 2025-10-28

**Authors:** Katerina Bernardi, Juan Darío Ortigoza-Escobar, Jana Dominguez-Carral, Iván Espinoza-Quinteros, Lorena Diaz Mendo, Anne Koy, Moritz Thiel

**Affiliations:** 1 Department of Pediatrics, Faculty of Medicine and University Hospital Cologne, University of Cologne, Cologne, Germany; 2 Department Human Neuroscience, Sapienza University, Rome, Italy; 3 Biomedical Network Research Centre on Rare Diseases (CIBERER), Instituto de Salud Carlos III, Madrid, Spain; 4 European Reference Network for Rare Neurological Diseases (ERN-RND), Barcelona, Spain; 5 Movement Disorders Unit, Department of Child Neurology, Institut de Recerca Sant Joan de Déu, Barcelona, Spain; 6 Epilepsy Unit, Department of Child Neurology, Institut de Recerca Sant Joan de Déu, Barcelona, Spain; 7 Pediatric Neurology Unit, Department of Pediatrics, Universidad Peruana Cayetano Heredia, Lima, Perú; 8 Center for Rare Diseases, Faculty of Medicine and University Hospital Cologne, University of Cologne, Cologne, Germany

**Keywords:** GNAO1, Movement disorder, Stridor, Tracheostomy, Upper airway management

## Abstract

**Background:**

*GNAO1*-related disorders (*GNAO1*-RD) encompass a wide phenotypic spectrum, including muscular hypotonia, movement disorders (MD), epilepsy, developmental delay, and intellectual disability. MD often presents with dystonia and choreoathetosis, and dyskinetic crises can lead to life-threatening conditions. Despite increasing reports, limited information exists on the impact of upper airway dysfunction in *GNAO1*-RD patients. This study examines the implications of muscular hypotonia on upper airway function and subsequent clinical outcomes.

**Methods:**

This study includes four patients, three from the *GNAO1* registry in Germany, with data collected from medical records including neurological examinations, EEG recordings, genetics, imaging studies, and video documentation of dyskinetic movements and respiratory symptoms. Treatment interventions and clinical outcomes were documented.

**Results:**

The study involved four patients (three males and one female) aged between 15 months and 12 years, all of whom were within the severe spectrum of *GNAO1*-RD. All patients exhibited severe hypotonia and hyperkinetic MD, leading to recurrent dyskinetic crises. Respiratory complications included an inspiratory stridor and airway obstructions. All patients died at young age (2.4, 2.8, 7.8 and 12 years) due to respiratory complications. Despite interventions such as DBS and tracheostomy, clinical outcomes remained poor.

**Conclusions:**

Upper airway dysfunction significantly contributes to the high morbidity and mortality in *GNAO1*-RD patients. Current therapeutic options are limited; while DBS can be life-saving during acute crises, it does not address swallowing or airway dysfunction effectively. Multimodal approaches and larger, multicenter trials are needed to improve outcomes for these patients.

**Supplementary Information:**

The online version contains supplementary material available at 10.1186/s13052-025-02150-0.

## Introduction

*GNAO1*-related disorders (*GNAO1*-RD) encompass a wide phenotypic spectrum, including muscular hypotonia, movement disorders (MD), epilepsy, developmental delay and intellectual disability [1, 2]. MD usually manifests during infancy, presenting with predominantly severe hypotonia combined with dystonia and choreoathetosis. Dyskinetic crises [3], or paroxysmal exacerbations of the MD, can occur spontaneously or be triggered by infections, pain, or emotional distress. These episodes can be life-threatening and are a cardinal feature of the disease in many patients [4]. Although milder and later-onset phenotypes have been identified [5], most reported cases exhibit a severe form of the disease, characterized by recurrent dyskinetic crises and substantial impairment in motor function, cognition, speech, swallowing, and sleep [6].

*GNAO1*-RD patients face a notable risk of mortality during early childhood [7], often due to refractory epilepsy or dyskinetic crises. Recurrent respiratory tract infections and severe dysphagia significantly increase the risk of aspiration, leading to significant clinical deterioration in these patients [8]. Another potential cause of respiratory dysfunction may be autonomic respiratory control dysfunction or obstructive sleep apnoea (OSA), as seen in other patients with epileptic encephalopathies. Dysfunction of the autonomic nervous system can interfere with the normal regulation of breathing, leading to issues such as apnea, hypoventilation, and irregular breathing episodes [9, 10]. Despite an increasing number of case reports and a broader understanding of the clinical spectrum of *GNAO1*-RD, there remains limited information on the impact of upper respiratory tract dysfunction in these patients. Therefore, this study aims to examine the implications of muscular hypotonia on upper airway function and subsequent clinical outcomes.

## Methods

Three out of the four patients are enrolled in the *GNAO1* registry in Germany, approved by the Ethics Committee of the Medical Faculty of the University of Cologne under identification number 19–19280. The parents of one patient residing outside Germany retrospectively consented to the publication of the case of their child. Prior to study participation, all participants gave informed consent. Clinical data were collected from medical records, including detailed histories, neurological examinations, electroencephalogram (EEG) recordings, genetics, and imaging studies where available. Video documentation of dyskinetic movements and respiratory symptoms, such as inspiratory stridor, was also reviewed. Treatment interventions, such as pharmacological management and surgical procedures, and clinical outcomes were documented. All patients underwent laryngotracheoscopy and bronchoscopy under spontaneous breathing.

## Results

Case 1 was a male patient who manifested with generalized seizures at the age of six weeks, with rapidly increasing frequency from thereon. A genetic panel for Early Infantile Epileptic Encephalopathy (EIEE) revealed a missense variant in *GNAO1*. He showed developmental delay and severe muscular hypotonia with a lack of head control. At six months of age, he manifested choreoathetosis of the limbs and persistent orofacial dyskinesia. Dystonia was present in the trunk, leading to recurrent opisthotonos. Apart from EIEE, a profound feeding problem due to severe dysphagia was prominent. At the age of eight months, he developed an increasing inspiratory stridor in the supine position with recurrent oxygen desaturation episodes (Supplementary Material 2). A laryngotracheoscopy with a rigid laryngoscope was performed under spontaneous breathing (Supplementary Material 3). The examination did not reveal any structural alterations or tracheo-laryngeal narrowing, suggesting that the stridor was secondary to muscular hypotonia of the upper airways. Due to a lack of therapeutic options, he was dependent on continuous head fixation in an upright position and oxygen therapy to improve his breathing. Unfortunately, the patient’s clinical conditions deteriorated constantly due to recurrent infections of the upper airways, with dyskinetic crisis necessitating numerous hospitalizations. Colonization of the nasal and throat regions with Klebsiella pneumoniae and 3MRGN Enterobacter was detected. In the course of a further severe respiratory infection, the patient died due to combined pulmonary and renal failure in a palliative outpatient setting.

Case 2 was a 15-month-old male with neurodevelopmental encephalopathy and MD due to a *GNAO1* de novo variant. Since birth, he experienced intermittent dyskinetic movements of the limbs with tongue protrusion. At the age of 4 months, the MD became aggravated by the onset of paroxysmal choreoathetosis and dystonia of the limbs and trunk with opisthotonos, general irritability, and excessive drooling. He suffered from several episodes of daily dyskinetic crises (0–8 episodes per day) with hyperkinesia of the limbs and the head, which worsened with stress and tactile stimulation. He had never any seizures; his psychomotor development was delayed with no motor milestones reached and impaired cognition. In the following, he was suffering from continuous hyperkinetic involuntary movements accompanied by severe central muscular hypotonia. Treatment with clonazepam led to a remarkable improvement in the MD. At the age of twelve months, he manifested an inspiratory stridor. In the following weeks, his respiratory condition deteriorated continuously, leading to a hospital admission and requiring respiratory support through noninvasive ventilation. The laryngotracheoscopy revealed moderate pharyngo-tracheomalacia, muscular hypotonia of the upper airways, and adenoid hypertrophy. Treatment with local steroids was started to reduce the hypertrophy, but the stridor persisted. In the following, he experienced recurrent respiratory infections with hospital admission, and deceased due to a severe pneumonia without detection of pathogens.

Case 3 was a male patient, born spontaneously at term. He manifested muscular hypotonia with a profound developmental delay. During the first year of life, the boy developed a severe hyperkinetic MD comprising dystonia and generalized choreoathetosis, with recurrent life-threatening dyskinetic crises requiring admission to the intensive care unit. Due to a persistent, severe dyskinetic crisis, Deep Brain Stimulation of the globus pallidus internus (Gpi-DBS) was performed as an emergency intervention as well as a tracheostomy at the age of eight years. Thereafter, the dyskinetic crisis stopped, and the MD improved. In the genetic testing, the missense variant in *GNAO1* could be identified. In the following days, he manifested a stridor at rest, which increased with the deterioration of the MD. Despite the tracheostomy, obstructions of the airways continued to occur, originating in the lower airways, which led to multiple hypoxic spells requiring reanimation. He was diagnosed with severe dynamic trachea-bronchomalacia, characterized by recurrent total collapse of the left main bronchus. Pseudomonas aeruginosa sensitive to ciprofloxacin was identified in the tracheal secretions. Permanent continuous positive airway pressure (CPAP) ventilation was not tolerated and led to exacerbations of the MD. The tolerable oxygen flow therapy could not stop the obstructive apnea, and the boy died at the age of 12 in a nursing home.

Case 4 Starting from the third day of life, this girl presented with focal seizures and subsequently a status epilepticus. Development was delayed, and abnormal hyperkinetic movements were observed since the first months of life. Furthermore, intermittent muscular hypotonia and sleep disturbances were prominent. Due to severe feeding problems and swallowing difficulties due to muscular hypotonia, a gastrostomy and later a jejunal feeding tube were performed. Exome analysis revealed a missense *GNAO1 de novo* variant. The movement disorder worsened over time, with recurrent severe dyskinetic crises. As a result, she underwent Gpi-DBS implantation at the age of three years, with an initial improvement in hyperkinesia. However, the girl started to exhibit significant impairment due to frequent vomiting of mucous secretion, often resulting in choking episodes. She experienced recurrent episodes of upper airway obstruction, likely due to mucous obstruction due to weak coughing in the context of muscular hypotonia, impaired coordination of swallowing, irregular, involuntary tongue movements, and excessive drooling. After several unsuccessful therapeutic approaches, including glycopyrronium bromide, anticholinergic drugs, dopamine antagonists, and botulinum toxin injections in the parotid gland, a tracheostomy was performed to protect her from aspirations of mucous secretions due to severe dysphagia. With the tracheostomy, there were no further aspirations, but vomiting persisted, and the need for mechanical suction increased. At the age of five, she underwent a fundoplication surgery with beneficial effects on the frequency of vomiting and hypersalivation. A colonization with 3MRGN Escherichia coli of the tracheal secretions was found. Death occurred at the age of 7.8 years due to acute respiratory distress syndrome (ARDS) resulting from a an Influenza A infection.

Table 1 resumes genetic findings, clinical and radiological features of the four patients.

Severity score is attached in Supplementary Material 1.

**Table 1:** Genetic variants and clinical features of the patients

Pat	Gen.Variant in GNAO1 reference sequence: NM_138736.3	Respiratory Symptom (onset in m) and treatment	Epileptic symptoms (ILAE classification of seizures 2017) and treatment	EEG	Movement disorder and treatment	Speech and feeding	Imaging	GNAO1-RD Severity score	Outcome	First published
P1	c.607G > A/ p.G203R	Stridor (8), MH of the UA. Head fixation in upright position and oxygen therapy	Neo: generalised motor onset; later focal and generalised motor onset; LEV: frequency reduction.	(6 m) no pathologic findings	DY trunk, mouth, MH, nystagmus. DC CZP -> DY+, GAB increased alertness, CL –> on demand	Anarthria; PEG	MRI (1 m): no pathologic findings	11,25	Deceased (2.4 y)	Thiel et al., 2022
P2	c.607G > A/ p.G203R	Stridor (12), pharyngo-tracheomalacia, MH of the UA and adenoid hypertrophy Local corticosteroids, non-invasive ventilation	None	(7 m and 15 m) no pathologic findings	DY limbs, trunk, CH, DC; MH BZD -> DY+, GAB ineffective	Anarthria; PEG	MRI (7 m) no pathologic findings	8,75	Deceased (2.8 y)	Domínguez-Carral, Jana et al. 2023
P3	c.607G > A/ p.G203R	Stridor (96), severe dynamic trachea-bronchomalacia tracheostomy, CPAP ventilation	None	(8 y) no pathologic findings	DY generalised; CH; MH; DC; Nystagmus THP: partial effect Baclofen: DY+; BZD & CL–> on demand Cannabis short effect; DBS(8 y): + on CH; no more DC	Dysarthria; PEG	MRI (72 m): EV; pseudotumour cerebri	8,5	Deceased (12 y)	Thiel et al., 2022
P4	c.170 A > C/ p.H57P	Stridor (3) MH of the UA, severe dysphagia; involuntary tongue movements, excessive drooling & repeated vomiting of mucus. GB, anticholinergic drugs, DA and botulinum toxin injections in the parotid gland: all ineffective. Tracheostomy & fundoplication -> reduction vomiting and hypersalivation	Neo: focal motor onset; 3 y SE; later no SZ anymore; CZP, TPM: frequency reduction	(3 y) no pathologic findings	Generalised DY; increasing CH; MH BZD -> DY+; Baclofen & GAB ineffective; CL on demand DBS(36 m): + on DY and CH, no more DC	Anarthria; PEG	CT(40 m): AT MRI(36 m): AT, EV	9,75	Deceased (7.8y)	Thiel et al., 2022

+: Positive effect, *AT* cerebral and cerebellar atrophy, *BZD* benzodiazepines, *CH* Chorea, *CL* Chloral hydrate, *CZP* Clonazepam, *DA* dopamine antagonists, *DBS* Deep brain stimulation, *DC* Dyskinetic crisis, *DY* Dystonia, *EV* Enlarged Ventricles, *GAB* Gabapentin, *GB* Glycopyrronium bromide, *LEV* Levetiracetam, *m* Months, *MD* Movement disorder, *MH* Muscular hypotonia, *neo* Neonatal, *PEG* percutaneous endoscopic gastrostomy, *SE* Status epilepticus, *SZ* Seizures, *THP* Trihexyphenidyl, *TPM* Topiramate, *UA* Upper respiratory airways, *y* Years

## Discussion

The four cases presented in this study illustrate the severe end of the phenotypic spectrum of *GNAO1*-RD, characterized by profound motor impairment and global developmental delay and all died at a young age. Three of the patients carried the c.607G > A/p.G203R pathogenic variant, which is known to be associated with the most severe phenotypes.

Severe hyperkinetic MD, leading to recurrent dyskinetic crises, is a hallmark feature of *GNAO1-RD* and a significant predictor of mortality [11–13]. The patients described exhibited pronounced hyperkinetic MD with distinctive orofacial involvement, impaired tongue coordination, and swallowing difficulties. In addition, muscle hypotonia and impaired postural control, which are early indicators of GNAO1-RD, contributed to the life-threatening respiratory distress [14].

The management of both hyperkinesia and central hypotonia poses significant challenges. Pharmaceutical treatment for dystonia and choreoathetosis can exacerbate resting muscular hypotonia [15], while hypersalivation, a common side effect of benzodiazepine therapy, further impairs upper airway function [16]. DBS has shown beneficial effects primarily on limb hyperkinesia in *GNAO1*-RD [8], yet its impact on orofacial hyperkinesia and hypotonia remains limited [14].

Upper airway dysfunction in all the described patients led to profound discomfort and frequent hospitalizations. The therapeutic approach involved multidisciplinary assessments, including laryngotracheoscopy, to identify treatable causes, consistent with guidelines from the International Pediatric Otorhinolaryngology Group (IPOG) for laryngomalacia [17]. Laryngeal dystonia (LD) with inspiratory stridor, potentially treatable with antidystonic medications or botulinum toxin injections into the vocal cords, was considered a differential diagnosis. This condition has been documented in cerebral palsy [18], and it can be hypothesized that it could cause stridor in *GNAO1-RD*. However, a definite diagnosis often requires fiberoptic laryngoscopy in an awake patient to detect vocal cord adduction during inspiration in the presence of generalized dystonia, as examination under anesthesia may obscure the pathophysiology of the stridor.

Nevertheless, there is considerable overlap between the patterns of inspiratory stridor in laryngomalacia and LD. In both conditions, inspiratory stridor worsens when patients are agitated and improves when they are relaxed. In this context, one may speculate that in case 3, overlapping dystonic airway obstructions may have prevented the patient from benefiting from a tracheostomy. Laryngomalacia, characterized by hypotonic narrowing of the upper airways, emerged as a major contributor to stridor and upper airway dysfunction in *GNAO1*-RD patients. Interestingly, early stridor onset has been linked to reduced survival in other conditions marked by hypotonia, such as multiple system atrophy [19].

In addition to upper airway disorders, lower airway abnormalities, central control of ventilation, autonomic dysfunction, and sleep-related breathing disorders can contribute to respiratory dysfunction in complex neuronal disorders [20]. To examine these features in GNAO1 might be of future relevance.

Brain structural deficits may be linked to disruptions in respiratory rhythm generation, as seen in chronic obstructive pulmonary disease and OSA [21, 22]. Regional changes in gray matter have been correlated with autonomic dysfunction in Parkinson’s disease (PD) [23] and autonomic failure has been proposed as a contributing factor to respiratory dysfunction in PD [24] and in patients with epileptic encephalopathies [8]. All but one of the patients discussed above had normal brain imaging, and no gray matter changes could be assessed in any of them. To assess this hypothesis in the future in *GNAO1*-RD, advanced brain imaging methods should be performed in larger cohorts, and polysomnographic evaluations could be useful to rule out the presence of sleep-related respiratory disorders.

Here we underscore the high morbidity and mortality associated with upper airway involvement in severely affected *GNAO1*-RD patients. Current therapeutic options are limited; while Gpi-DBS may be life-saving during acute dyskinesis crises, it has no proven efficacy in managing swallowing or upper airway dysfunction [25]. Respiratory muscle therapy through targeted speech interventions has shown promise in alleviating obstructive sleep apnea related to hypotonia [26], but its application in severely affected patients remains challenging. Tracheostomy was performed in cases 3 and 4, and although it did not uniformly yield beneficial outcomes, in severe affected patients, such as those with c.607G > A/p.G203R pathogenic variant, an early invasive approach could be considered to prevent the progressive deterioration of the airway function.

This study is limited by its small sample size of only four patients, which may not fully capture the variability of the *GNAO1*-RD spectrum. Additionally, the retrospective nature of the case reviews and the reliance on medical records in cases 2 and 3 may introduce bias or incomplete data.

## Conclusion

We conclude that upper airway involvement in severely affected *GNAO1-RD* patients should not be underestimated and may serve as a predictor of reduced survival, exacerbated by the current lack of effective therapies. Clinicians should adopt a multimodal approach involving diverse specialties and diagnostic tools like laryngotracheoscopy to address this critical aspect of patient care. Future research efforts should prioritize larger, multicenter prospective trials to enhance the management and outcomes of these complex, disabled patients with *GNAO1*-RD.

## Supplementary Information

Supplementary Material 1.

Supplementary Material 2.

Supplementary Material 3.

Supplementary Material 4.

## Data Availability

The complete datasets generated during and/or analysed during the current study are not publicly available since only relevant data concerning the scientific question of this study is included, but datasets are available from the corresponding author on reasonable request.

## Abbreviations

+: Positive effect
ARDS: Acute respiratory distress syndrome
AT: Cerebral and cerebellar atrophy
BZD: Benzodiazepines
CH: Chorea
CL: Chloral hydrate
CPAP: Continuous positive airway pressure
CZP: Clonazepam
DA: Dopamine antagonists
DBS: Deep brain stimulation
DC: Dyskinetic crisis
DY: Dystonia
EEG: Electroencephalogram
EIEE: Early Infantile Epileptic Encephalopathy
EV: Enlarged Ventricles
GAB: Gabapentin
GB: Glycopyrronium bromide
GNAO1-RD: GNAO1-related disorders
Gpi-DBS: Deep Brain Stimulation of the globus pallidus internus
IPOG: International Pediatric Otorhinolaryngology Group
LD: Laryngeal dystonia
LEV: Levetiracetam
m: Months
MD: Movement disorder
MH: Muscular hypotonia
neo: Neonatal
OSA: Obstructive sleep apnoea
PD: Parkinson’s diseas
PEG: Percutaneous endoscopic gastrostomy
SE: Status epilepticus
SZ: Seizures
THP: Trihexyphenidyl
TPM: Topiramate
UA: Upper respiratory airways
y: Years
