# Supplementary material for: Severe upper airway dysfunction in GNAO1-related disorders
Source: Ital J Pediatr. 2025 Oct 28;51:296. doi: 10.1186/s13052-025-02150-0 (PMC12570824; doi:10.1186/s13052-025-02150-0)
Supplement: Supplementary file 4 — Supplementary Material 4. [file 13052_2025_2150_MOESM4_ESM.docx]

Professor Angelo Selicorni, MD, UOC Pediatria ASST Lariana, Centro Fondazione Mariani per il Bambino Fragile, Como Italy
*Editor, Italian Journal of Pediatrics*

13 August 2025

Dear Professor Selicorni,

We would like to express our sincere gratitude for sending us the reviewed version of our article **“Severe upper airway dysfunction in GNAO1-related disorders” (ITJP-D-24-00773).**

We are very pleased to learn that our manuscript is considered potentially acceptable for publication. We are truly thankful for the constructive and thoughtful comments provided by the reviewer, which have been invaluable in improving the quality and clarity of our work. We have carefully addressed each of the reviewers’ suggestions and believe that these revisions have strengthened the manuscript and improved readability.

For your convenience, we have attached a detailed, point-by-point response to all reviewer comments. Additionally, we have uploaded a revised version of the manuscript with tracked changes, so that all modifications are clearly visible.

We would also like to inform you that we have updated the clinical information for one patient included in the study. Sadly, the young girl passed away in the interim due to a pulmonary infection. This unfortunate event further underscores the high mortality risk among young patients with upper airway dysfunction in GNAO1-related disorders.

Once again, we sincerely thank you for your time, consideration, and support in the review process of our manuscript.

Sincerely yours

Moritz Thiel, MD (Corresponding Author)*.*
